# Computational Investigation of the Monomer Ratio and Solvent Environment for the Complex Formed between Sulfamethoxazole and Functional Monomer Methacrylic Acid

**DOI:** 10.1021/acsomega.2c00862

**Published:** 2022-05-10

**Authors:** Sisem Ektirici, Önder Kurç, Mitra Jalilzadeh, Süleyman Aşır, Deniz Türkmen

**Affiliations:** † Department of Chemistry, Faculty of Science, Hacettepe University, Beytepe, Ankara 06800, Turkey; ‡ Department of Materials Science and Nanotechnology Engineering, Near East University, Nicosia 99138, Mersin 10 Turkey, North Cyprus

## Abstract

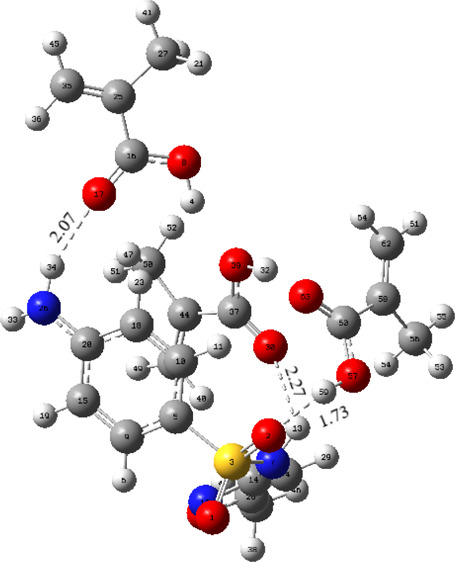

In this study, the molecularly imprinted polymers (MIPs) that will be formed by the sulfamethoxazole (SMX) molecule and methacrylic acid (MAA) molecule were examined theoretically. The most stable interaction region between the two molecules was determined in solvent environments (ethanol, acetonitrile, and dimethylsulfoxide), and monomer ratios (SMX/MAA; 1:1, 1:2, and 1:3) were examined to form the most stable geometry. The number and length of the hydrogen bonds formed between the template molecule and the functional monomer and the interaction between the atoms were determined. Geometry optimizations of the molecules were calculated by the DFT method at the M06-2X/ccpVTZ level, and single-point energy calculations were carried out at the B2PLYP-D3/ccpVDZ level. In addition to the theoretical studies, the experimental Fourier-transform infrared spectroscopy (FTIR) spectrum of the complex formed between SMX and MAA was compared with the theoretical FTIR spectrum. As a result of the studies, the monomer ratio and solvent environment in which the stable complex was formed were determined in the MIP studies carried out with the SMX template molecule and MAA monomer. The most stable template molecule–monomer ratio of the complex between SMX and MAA was determined to be 1:3, and the solvent medium in which the most stable geometry was formed was acetonitrile.

## Introduction

It has been shown that the industrial production of antibiotics and their use in animals pollute water and fauna. It also increases antibiotic resistance and exposure to antibiotic residues, resulting in hypersensitivity, carcinogenicity, mutagenicity, teratogenicity, bone marrow depression, and disruption of normal intestinal flora.^1−3^ The Organization for Economic Co-operation and Development estimates that antibiotic resistance has killed approximately 700,000 people globally. This number could reach 9.5 million if the current resistance level increases by 40%.^4^ Those reports show an environmental and health risk posed by antimicrobial residues. To prevent the danger they possess, the regulatory agencies of most countries have evaluated their toxicological data and determined the maximum residue limit (MRL).

Tetracycline, penicillin, fluoroquinolones, and sulfonamides are veterinarians’ most commonly used antibiotics.^5^ Sulfonamides are an antibiotic family well known for their low production price and effectiveness as a therapeutic agent. Sulfonamides interfere with the folic acid synthesis pathway of bacteria and stop their growth and proliferation. Sulfamethoxazole (SMX) is one of the sulfonamide antibiotics. SMX is widely used in treating urinary tract infections, gastrointestinal infections, respiratory infections, and trimethoprim in humans and animals. SMX with other antibiotics is given to patients with HIV and other immunologic-protective measurements against opportunistic infections.^6^ Their usage in large quantities, being widely used in animal and human treatment, increases the risk of environmental pollution and, therefore, antibiotic resistance.^7^ Its presence has been reported in sewage sludge,^8^ milk, and other food samples from various communities, which show a high residual rate,^9^ and freshwater^10,11^ in various countries.

For SMX, according to the European Medicinal Agency, the Committee for the Veterinary Medicinal Product (EMEA/MRL/026/95) evaluated the sulfonamide group antibiotic’s toxicology. It determined 100 μg/kg MRL for sulfonamide group residues in edible animal tissues. Most of the time, these agencies work with local governments to enforce MRL limits on foods and water and detect their presence in those sources that require good detection techniques.

Molecular imprinting is a candidate for the detection of antibiotic residues and purification. Depending on the product type, molecularly imprinted polymers (MIPs) may have a very high mechanical durability resistance against heat, pressure, acids, bases, and organic solvents. Due to their stability in extreme conditions, MIPs have been drawing the attention of many industries, like pharmaceuticals.^12−16^ Because of the properties of MIPs, their use in antibiotic detection in food and environmental samples, which have gained importance from the study areas of biochemical molecules, is also essential. However, their properties are directly affected by important parameters^17,18^ such as monomer–template type, interaction strength between them, and solvent type, which requires serious planning before synthesizing MIPs. Investigating these parameters involves many laborious tests^17,19^ and experiments.

Theoretical studies, before the wet experiments of biological molecules, are essential in terms of time, cost, and efficiency.^20−22^ Although the first molecular mechanics (MM) methods were used to examine the interactions of molecules computationally, researchers have been using quantum mechanical (QM) methods more and more in the last decade. Although QM offers more accurate results than MM, QM methods rely more on computational power due to the number of calculations needed. To use this method, many techniques are employed to shorten its calculation, such as the use of machine learning^23−26^ by taking advantage of its learning algorithms and shortening process, or using methods like density functional theory (DFT) and reducing the amount of calculations, these methods shorten the required computational cost and could make computational monomer screening more available; DFT and becke 3-parameter exchange correlation function (B3LYP) hybrid theory and Minnesota 06 (M06-2X) is a well-known and versatile technique used in computational chemistry for investigating the interaction between molecules.^27−29^ It is possible to employ this technique to optimize some aspects of MIP effects^30,31^ without laborious experiments.

Within the scope of the study, to establish a theoretical basis for MIP applications, the interactions of the SMX molecule, one of the antibiotics that cause antibiotic residues and environmental pollution, with the functional monomer methacrylic acid (MAA) were examined theoretically, and a pioneering study was conducted for the experimental studies to be carried out. The monomer ratio and solvent environment of the most stable complex formed between the two molecules were determined. The most stable geometry (optimized geometry), intermolecular hydrogen bonds, interaction energies, Gibbs free energies, highest occupied molecular orbital–lowest unoccupied molecular orbital (HOMO–LUMO) energy, and energy gap were calculated. Problems such as the monomer ratio and solvent environment, which are of great importance for MIP applications, contributed to time and efficiency with the results obtained in this theoretical study for SMX and MAA molecules. Molecular geometries at different binding sites, as a measure of stability, interaction energies, Gibbs free energies, and hydrogen bonds were investigated with the M06-2X method ccpVTZ basis set. The HOMO–LUMO energies, HOMO–LUMO energy gaps, experimental Fourier-transform infrared spectroscopy (FTIR) spectra, and theoretical FTIR spectra of the most stable complex were compared. The complexes’ interaction energies and hydrogen bonds were investigated at 1:1, 1:2, and 1:3 monomer ratios for the most stable complex formation. The interactions between the template molecule and functional monomer were investigated in ethanol, acetonitrile, and dimethylsulfoxide (DMSO) solvents.

## Materials and Methods

### Materials

Geometry optimizations and single point energies of all molecules were calculated with the Gaussian 09 program. Molecular structures and theoretical FTIR spectra (obtained) were visualized with the Avogadro 1.2 program. The template molecule SMX (analytical standard) and monomer MAA used for FTIR studies were obtained from Sigma-Aldrich. Solvent DMSO, acetonitrile, and ethanol were supplied from Merck. Experimental FTIR characterization was done by an FTIR–attenuated total reflection spectrophotometer (Thermo Ficher Scientific, Nicolet is5, Waltham, USA) in the 400–4000 cm^–1^ range of wavenumber with a resolution of 2 cm^–1^.

## Methods

Template molecule SMX, functional monomer MAA, and template molecule–monomer complex geometries were first modeled with the Gaussview 5.0 program. Geometries obtained after pre-optimization were subjected to geometry optimization with the DFT method at the M06-2X^32^/ccpVTZ level.^33^ B2PLYP-D3/^34^cpVDZ calculations were used for the single point energies and thermochemical parameters. Dispersion effect was corrected with the Grimme’s dispersion model.^35^ The interaction energies of the molecules at different monomer ratios (Δ*E*),^36^ Gibbs free energies (Δ*G*) in different binding sites, and solvation energies in different solvents (Δ*E*_solvation_)^37,38^ were calculated by eqs 1–3, respectively, and all theoretical FTIR spectra were obtained with the Gaussian 09 program.

### Interaction Energies and Gibbs Free Energies in the Gas Phase

The interaction energy measures the stability of the complex geometries formed between the SMX template molecule and the MAA monomer. The lower the interaction energy of the complex, the greater the stability of the complex geometry and the interaction forces between SMX and MAA.^39^ For this reason, the gas-phase interaction energies of complexes with molar ratios of 1:1 [SMX–MAA] (Δ*E*_1_), 1:2 [SMX–MAA–MAA] (Δ*E*_2_), 1:3 [SMX–MAA–MAA–MAA] (Δ*E*_3_), and Gibbs free energies for different binding sites were calculated. The basis set superposition error (BSSE) corrected by using a counterpoise method (CP).^40,41^ Template molecules (SMX), monomers (MAA), and complexes of SMX with different MAA ratios were optimized and their single-point energies calculated.

The interaction energy of the complex consisting of template molecule, SMX, and the functional monomer, MAA, was calculated by eq 1.
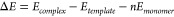
1

*E*_complex_ is the energy of the [SMX–MAA] complex, *E*_template_ is the energy of the SMX molecule, and *nE*_monomer_ is the energy of the MAA molecule that changes with the monomer ratio used.
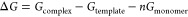
2

*G*_complex_ is the Gibbs free energy of the [SMX–MAA] complex, *G*_template_ is the Gibbs free energy of the SMX molecule, and *nG*_monomer_ is the total Gibbs free energy of the MAA molecules that changes with the monomer ratio used.

### Solvation Energies

In non-covalent imprinting applications, choosing the appropriate solvent for the non-covalent interactions between functional monomers and imprinted molecules for complex formation is very important for complex stability. The appropriate solvent can increase the efficiency of the prepared structures. In the process of preparing MIPs, the solvent must not only have the ability to dissolve all the component reagents in the reaction but also not interfere with the interactions between the template molecules and monomers. For the solvent effect, we used the solvation model based on density (SMD); SMD is a universal continuum solvation model applied for both charged and non-charged solute systems. To use the solvent model, we defined the molecular cavity with the Van der Waals surface cavity model and built a cavity using the universal force field.

For selecting the most suitable solvent that will show the highest efficiency for the imprinted polymers, the energies of the 1:1 M SMX–MAA complexes in ethanol, acetonitrile, and DMSO solvents were calculated by the M06-2X theory and ccpVTZ basis set.

Solvation energies (Δ*E*_solvation_) of the stable complexes in different solvents are calculated using eq 3.
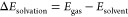
3

*E*_gas_ is the interaction energy of the SMX–MAA complex in the vacuum environment, and *E*_solvation_ is the interaction energy of the [SMX–MAA] complex in the solvent environment.

In addition to the interaction energies, Gibbs free energies are calculated according to eq 4 to investigate the most stable pre-complex between SMX and MAA molecules in the solvent environment.
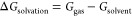
4

The thermodynamic scheme given for the solvent medium calculation is shown below.

### FTIR Analysis

As it is known, FTIR is a characterization method used for the identification and detailing of the functional groups in processes such as material identification and verification, copolymer evaluation, molecular fragmentation evaluation, basic drug research and structural explanation, formula development and validation, and quality control processes of materials. Also, FTIR is frequently used in MIP applications to correlate the computational results of template molecules, functional monomers, and complexes with the experimental results.^42−44^ This study compared the experimental and theoretical FTIR spectra of the most stable complex formed by SMX and MAA molecules.

## Results and Discussion

### Determination of Binding Sites

**Figure 1:**
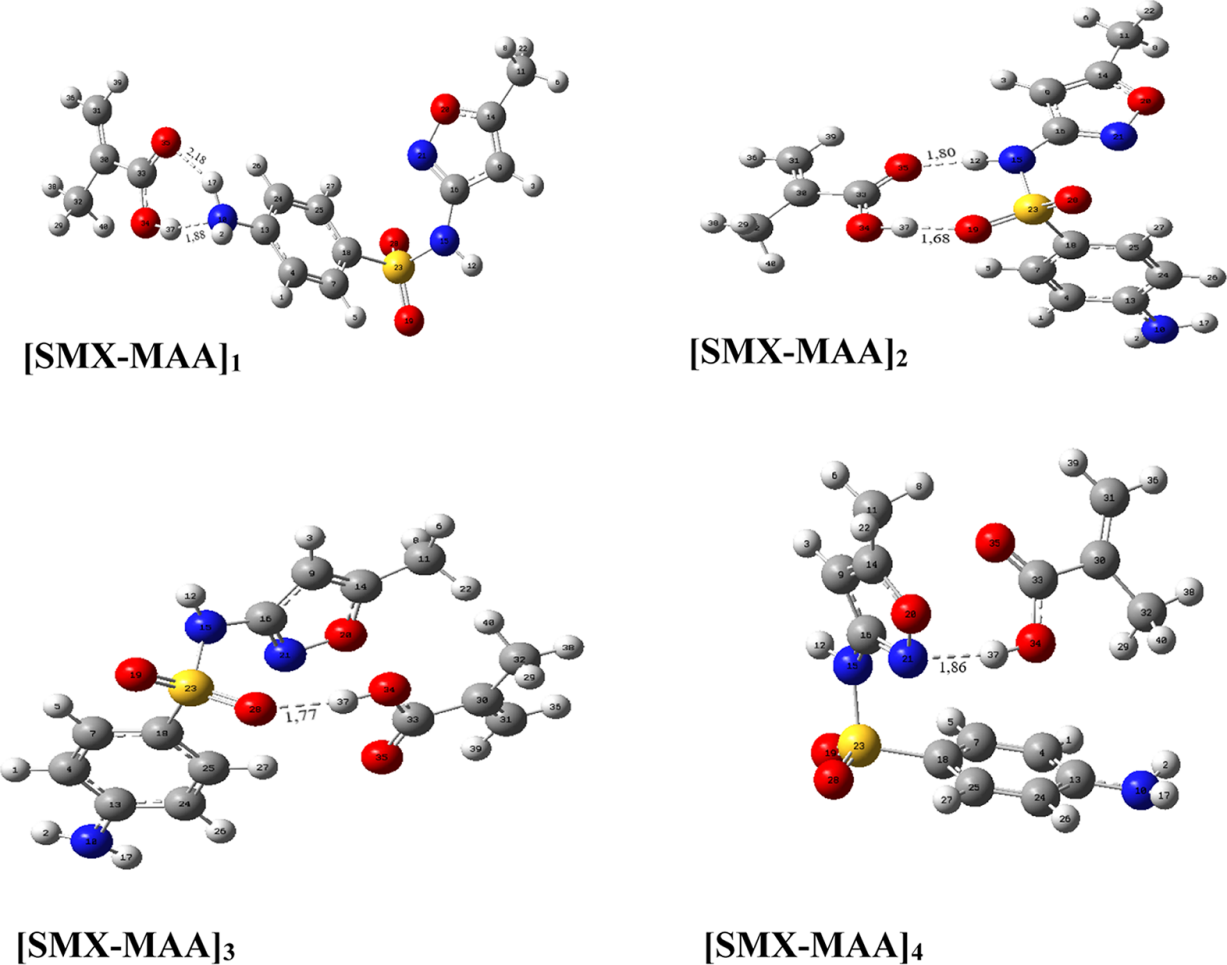
Hydrogen bonds and complex geometries formed between SMX and MAA at different binding sites.

**Scheme 1:**
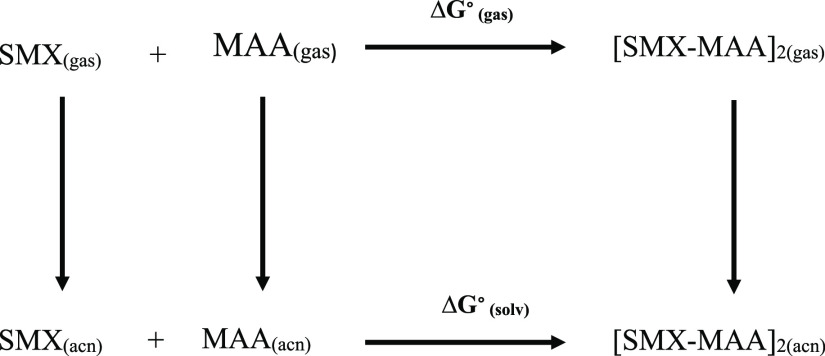
Schematic Representation of the Gibbs Free Energies for the Pre-Complexes of SMX and MAA Molecules in a Solvent Medium

For MIPs, hydrogen bond formation and the strength of the hydrogen bonds are essential in terms of stability and selectivity.^45^ Because the template molecule, SMX, contains many different interaction sites that can form hydrogen bonds with the monomer, MAA, it is important to determine which regions the monomer interactions are most effective. Therefore, M06-2X/ccpVTZ calculations were carried out for the interaction energies and formed hydrogen bonds at different potential binding sites at a molar ratio of 1:1, and after calculations, BSSE was corrected (Scheme 1). The interaction regions and hydrogen bonds of the optimized geometries are given in Figure 1. The interaction energies, Gibbs free energies, and number of hydrogen bonds formed are given in Table 1. Table 2 shows from which atoms the hydrogen bonds are formed and their distances.

**Table 1:** Interaction Energies, Gibbs Free Energies, and Number of Hydrogen Bonds Formed at the Different Binding Sites of [SMX–MAA] Complexes (kcal mol^–1^)

complexes	Δ*E*	Δ*G*	H bond
[SMX–MAA]_1_	–9.94	1.66	2
[SMX–MAA]_2_	–17.53	–5.37	2
[SMX–MAA]_3_	–10.57	–2.34	1
[SMX–MAA]_4_	–9.86	–2.41	1

As seen in Figure 1, many different regions can form hydrogen bonds between SMX and MAA molecules. The values of interaction energies of [SMX–MAA] complexes interacting from different binding sites are shown in Table 1. According to the data obtained from Table 1, the order of interaction energies of SMX–MAA complexes is [SMX–MAA]_4_ > [SMX–MAA]_1_ > [SMX–MAA]_3_ > [SMX–MAA]_2_. Because the hydrogen bonds in the [SMX–MAA]_4_ complexes are formed with the atoms in the rings of the SMX molecule, secondary interactions between the template molecule and monomer in the complex to be formed are not preferred from these regions. As can be seen from the Gibbs free energies calculated from eq 2, the complex with the only negative value appears to be attributed to the [SMX–MAA]_2_ complexes. In Tables 1 and 2, hydrogen bond interactions were observed in [SMX–MAA]_1_ and [SMX–MAA]_2_ complexes, and one hydrogen bond interaction was observed in [SMX–MAA]_3_ and [SMX–MAA]_4_ complexes. Based on the strength of the hydrogen bonds formed, it is seen that the strongest hydrogen bonds are formed in the [SMX–MAA]_2_ complexes.

**Table 2:** Hydrogen Bonds Are Formed between SMX and MAA Molecules and Their Lengths (Å)

complex	interacted atoms	bond length (Å)
[SMX–MAA]_1_	N10···H37	1.888
	O35···H17	2.183
[SMX–MAA]_2_	O35···H12	1.808
	H37···O19	1.683
[SMX–MAA]_3_	O28···H37	1.775
**[SMX–MAA]**_**4**_	**N21···H37**	**1.868**

### Frontier Molecular Orbitals of [SMX–MAA]_2_ Complex

It is well known that the HOMO and LUMO energies play a significant role in elucidating reaction mechanisms. HOMO energy measures a molecule’s ability to donate electrons, while LUMO energy is a measure of its ability to accept electrons. Because the [SMX–MAA]_2_ complex from different interaction regions constitutes the most stable geometry, HOMO–LUMO energies and HOMO–LUMO energy gap differences of the formed complex, the SMX template molecule and MAA monomer are given in Table 3 (in eV) for a detailed investigation of this complex. B2PLYLP-D3/ccpVDZ calculations were performed for the molecular orbital surfaces of the template molecule SMX, the functional monomer MAA, and the [SMX–MAA]_2_ complex, and the calculation results are given in Table 3.

**Table 3:** HOMO (*E*_HOMO_) and LUMO (*E*_LUMO_) Energies, and Energy Gaps (Δ*E*) of SMX, MAA, and [SMX–MAA]_2_ Molecules in eV

molecule	*E*_HOMO_ (eV)	*E*_LUMO_ (eV)	Δ*E* (eV)
SMX	–7.65	–4.18	3.47
MAA	–9.67	–4.58	5.09
**[SMX–MAA]**_**2**_	**–7.68**	**–4.58**	**3.10**

**Figure 2:**
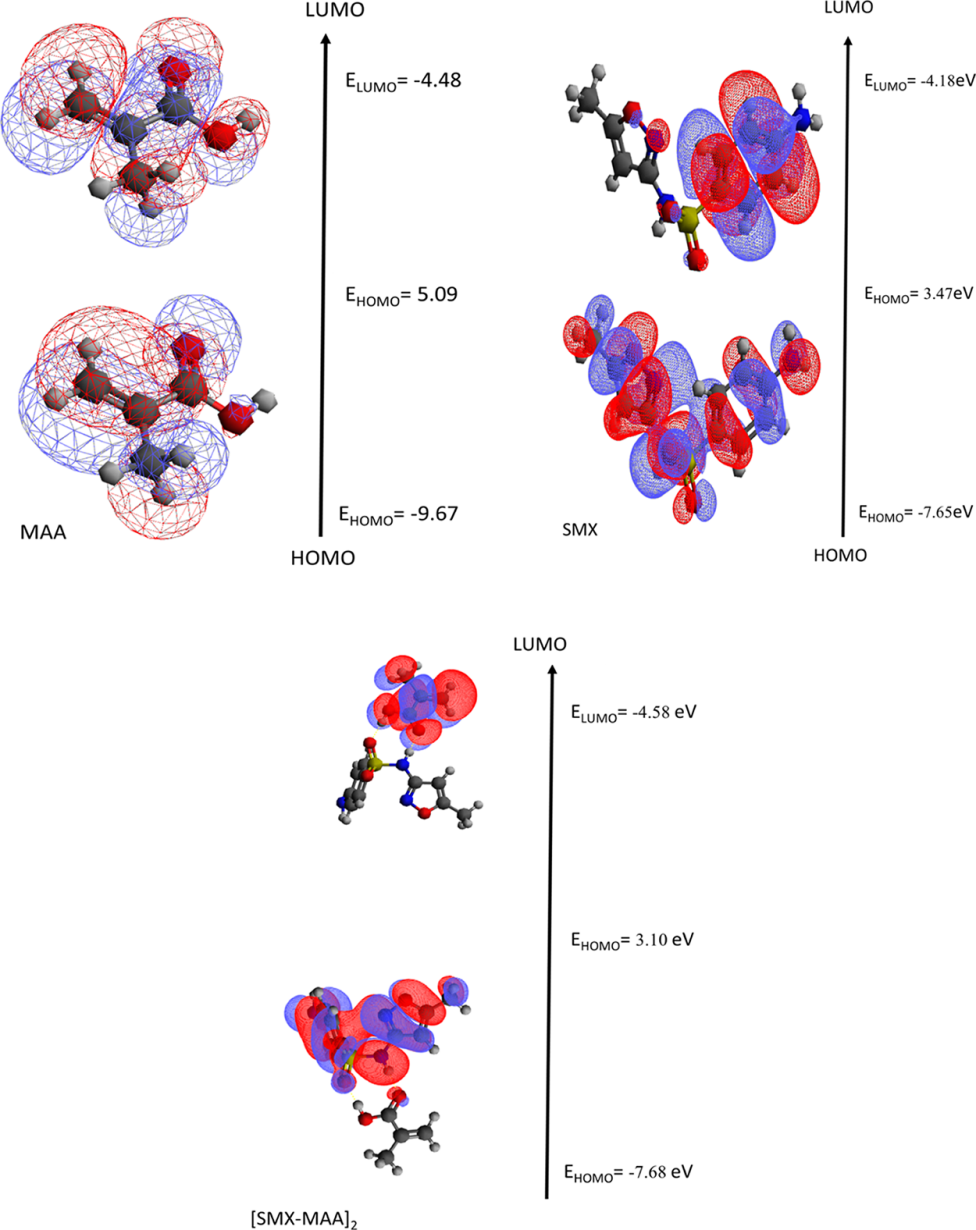
Molecular orbital surfaces and HOMO–LUMO energies (in eV) of the template molecule SMX, functional monomer MAA, and [SMX–MAA]_2_ complexes.

As seen in Table 3, the HOMO energy of the SMX molecule (*E*_HOMO_) was found to be greater than the HOMO energy of the MAA molecule. The LUMO energy of the MAA *E*_LUMO_ molecule was lower than the LUMO energy of the SMX molecule. In line with these data, the more active SMX template molecule is the electron donor, while the less active MAA monomer is the electron acceptor in the [SMX–MAA]_2_ complex (Figure 2).

### Determination of Molar Ratio

**Figure 3:**
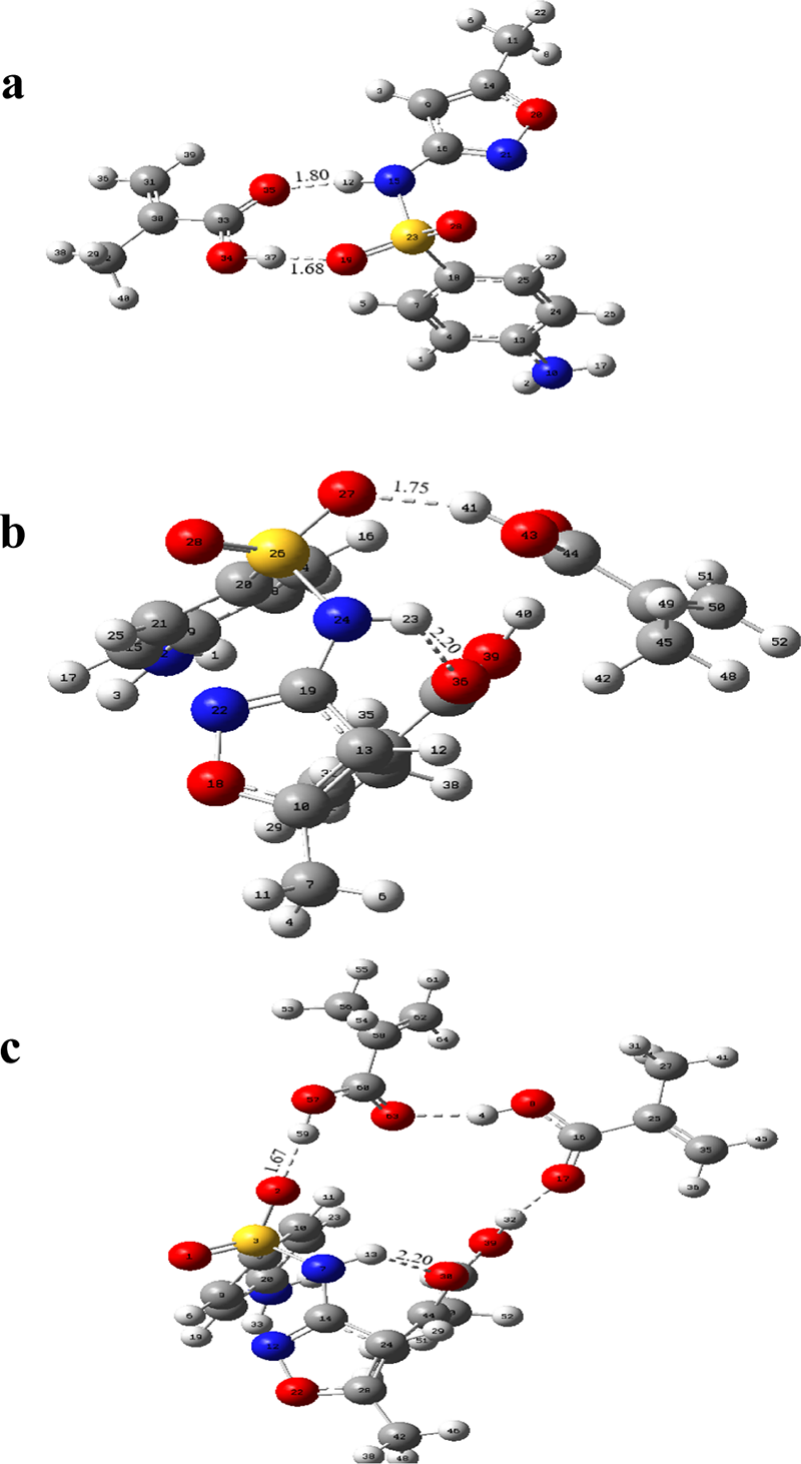
Optimized geometries and hydrogen bond lengths of the [SMX–MAA]_2_ complexes in the same binding site at molar ratios of (a) 1:1, (b) 1:2, and (c) 1:3.

Although the region that gives the most stable complex geometry at a 1:1 M ratio is calculated as the [SMX–MAA]_2_ complexes after the calculations above when the monomer molar ratio changes, the interaction energies and hydrogen bonds between the molecules are unknown. The interaction energies of the [SMX–MAA]_2_ complexes in molar ratios of 1:1, 1:2, and 1:3 were calculated to answer this question. The optimized geometries are given in Figure 3, and interaction energies are given in Table 4.

**Table 4:** Interaction Energies of [SMX–MAA]_2_ Complexes Interacting at Different Monomer Ratios; 1:1, 1:2, and 1:3

molar ratio	Δ*E* (kcal mol^–1^)
1:1	–17.53
1:2	–27.42
1:3	–37.94

As the complex’s interaction energy lowered, the complex geometry’s stability increased. The molar ratio at which the most stable geometry will be formed was calculated to be the [SMX–MAA]_2_ complex, which has the most stable binding interaction region. It was found that the most stable geometry would be formed at a molar ratio of 1:3 from interaction energies. The interaction energy of the [SMX–MAA]_2_ complexes in a 1:3 M ratio was calculated to be −37.94 kcal mol^–1^. Hydrogen bonds between SMX–MAA molecules were formed between O2–H57 and O38–H13 atoms, and the lengths of these bonds were determined to be 1.67 and 2.20 Å, respectively.

### Determination of the Solvent

**Figure 4:**
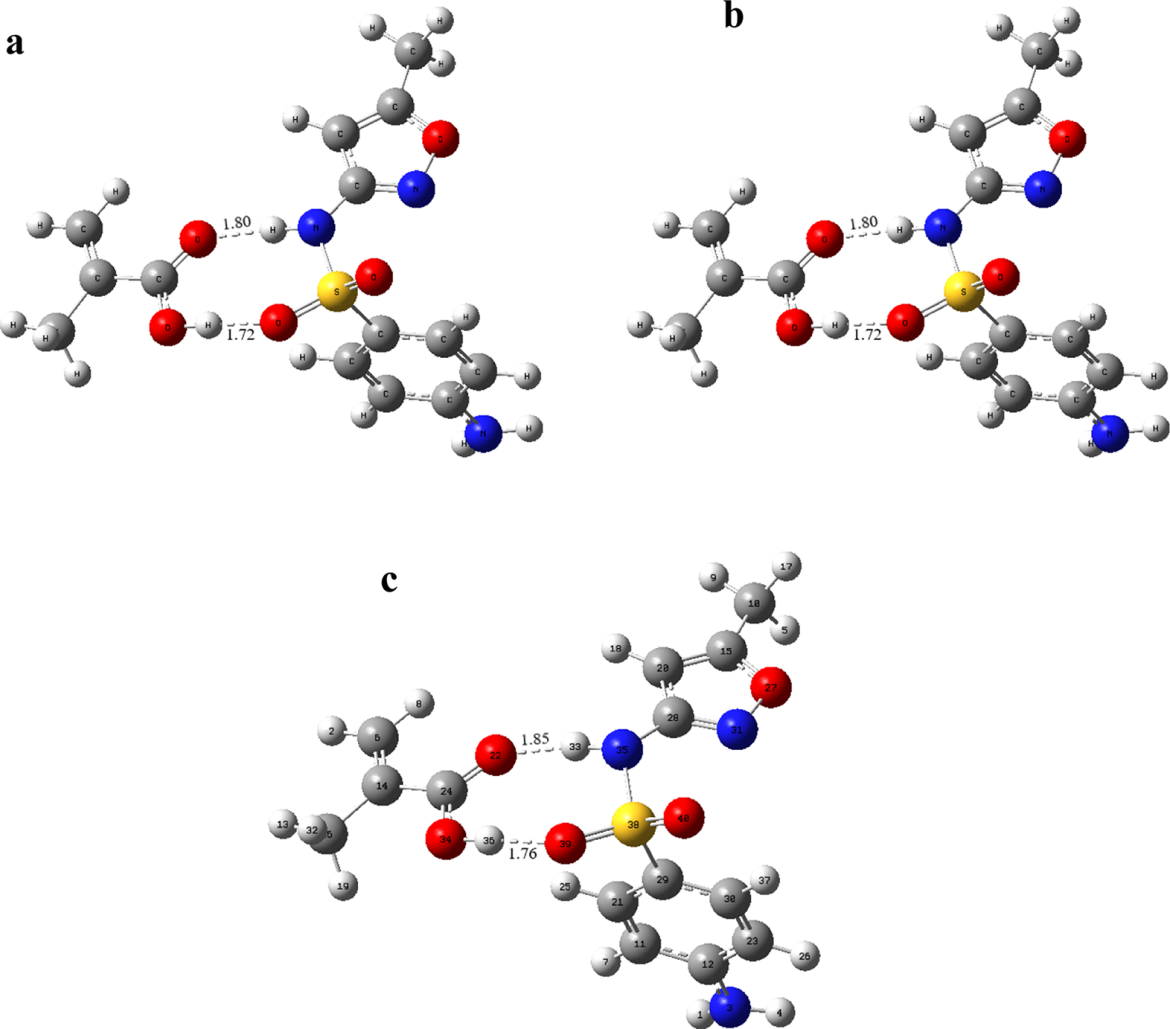
Interactions between the template (SMX) and the monomer (MAA) in different solvents (a: DMSO, b: ethanol, and c: acetonitrile) at a molar ratio of 1:1.

In non-covalent imprinting, it is crucial to screen solvents to form stable complexes with non-covalent interactions between functional monomers and imprinted molecules. For this reason, within the scope of the study, solvation energy (Δ*E*_solvation_) values of [SMX–MAA]_2_ complexes calculated in eq 3 are calculated for ethanol, acetonitrile, and DMSO solvents in a 1:1 M ratio using the DFT method at the B2PLYP-D3/ccpVDZ level. Table 5 shows the solvation energies Δ*E*_solvation_ (kcal mol^–1^) of SMX, MAA, and [SMX–MAA]_2_ molecules in ethanol, acetonitrile, and DMSO solvents. Table 5 shows the hydrogen bonding atoms and the lengths of these bonds in the [SMX–MAA]_2_ complexes in different solvents in a 1:1 M ratio (Figure 4).

**Table 5:** Solvation Energies (Δ*E*_solvation_) and Gibbs Free Energies of SMX, MAA Molecules, and [SMX–MAA]_2_ Complexes in Ethanol, Acetonitrile, and DMSO Solvents (kcal mol^–1^)

	ethanol		acetonitrile		DMSO	
molecule	Δ*E*_solvation_	Δ*G*_solvation_	Δ*E*_solvation_	Δ*G*_solvation_	Δ*E*_solvation_	Δ*G*_solvation_
SMX	–20.88	–19.67	–21.37	–20.64	–19.90	–19.00
MAA	–6.28	–5.74	–5.41	–5.30	–4.76	–4.68
[SMX–MAA]_2_	–12.62	1.10	–20.39	0.01	–18.44	0.58

Considering the Δ*E*_solvation_ energy values, the order of solvation energies for the [SMX–MAA]_2_ complexes, which has the most stable binding site, was calculated as ethanol > DMSO > acetonitrile. The order of Gibbs free energy values was calculated as ethanol > DMSO > acetonitrile. The hydrogen bond between the O25–H36 atoms given in Table 6 is calculated as 1.723 Å in the acetonitrile solvent, stronger than the hydrogen bonds formed between the same atoms in other solvents. The solvent with the best interaction between SMX and MAA was determined as acetonitrile based on the obtained results.

**Table 6:** Atoms Forming Hydrogen Bonds and Hydrogen Bond Lengths (Å) in [SMX–MAA]_2_ Complexes in Different Solvents

solvent	interacted atoms	bond length (Å)
ethanol	O35···H2	1.852
	O25···H36	1.760
acetonitrile	O35···H2	1.816
	O25···H36	1.723
DMSO	O35···H2	1.808
	**O25···H2**	**1.721**

### [SMX–MAA]_5_ Complex

**Figure 5:**
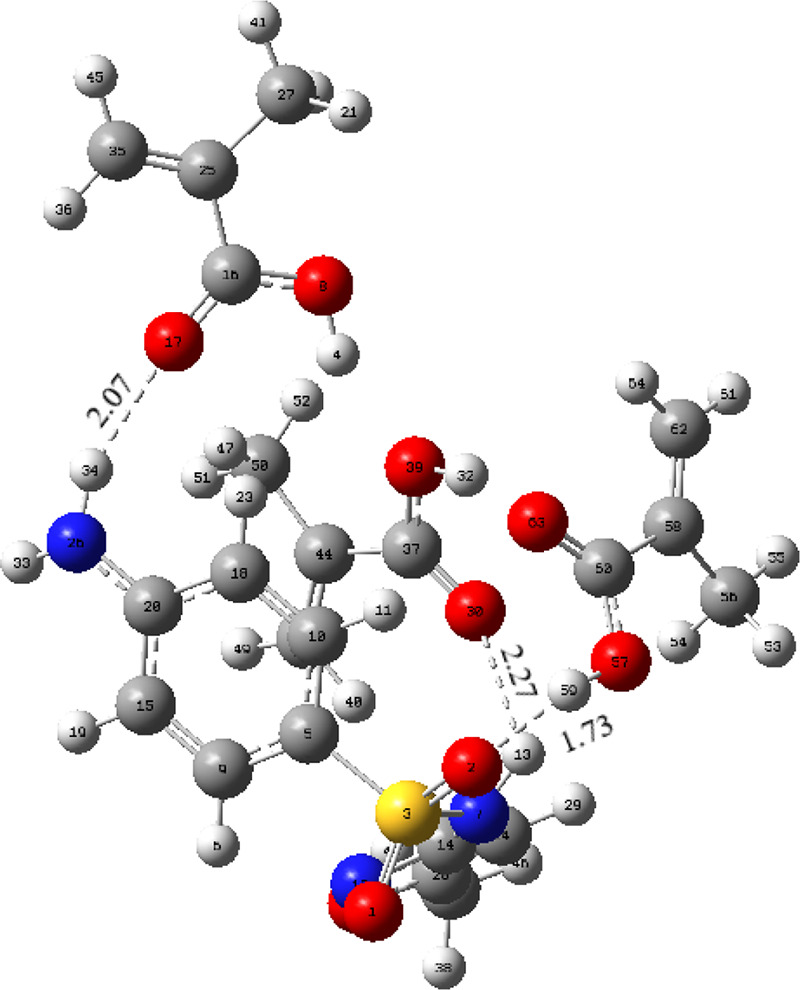
Optimized geometry of the [SMX–MAA]_5_ complexes.

Because the 1:3 M ratio is calculated on the same site in the complex with the most stable geometrical binding site, it is also possible for the monomers to bind from different sites in this determined molar ratio. In this possibility, interaction energy values were calculated at different binding sites of the [SMX–MAA] complex at a molar ratio of 1:3. Because [SMX–MAA]_1_ and [SMX–MAA]_2_ complexes give the lowest interaction energies, and the hydrogen bond strength in these complexes is the highest, the complex energies were calculated by including the binding sites of the two complexes at a molar ratio of 1:3; the complex geometry is given in Figure 5.

The hydrogen bonds in the molecule [SMX–MAA]_5_, which is the most stable of the geometries found between 3 MAA molecules and 1 SMX molecules, are formed between O30–H13 and O2–H50 H34–O17 atoms, and their lengths are calculated to be 2.27, 1.73, and 2.07 Å, respectively.

**Table 7:** HOMO–LUMO Energies (eV) and Energy Gaps of SMX, MAA, and [SMX–MAA]_5_ Molecules in the Gas Phase

molecule	*E*_HOMO_	*E*_LUMO_	Δ*E*
SMX	–7.65	–4.18	3.47
MAA	–9.67	–4.58	5.09
[SMX–MAA]_5_	–7.74	–4.64	3.10

**Figure 6:**
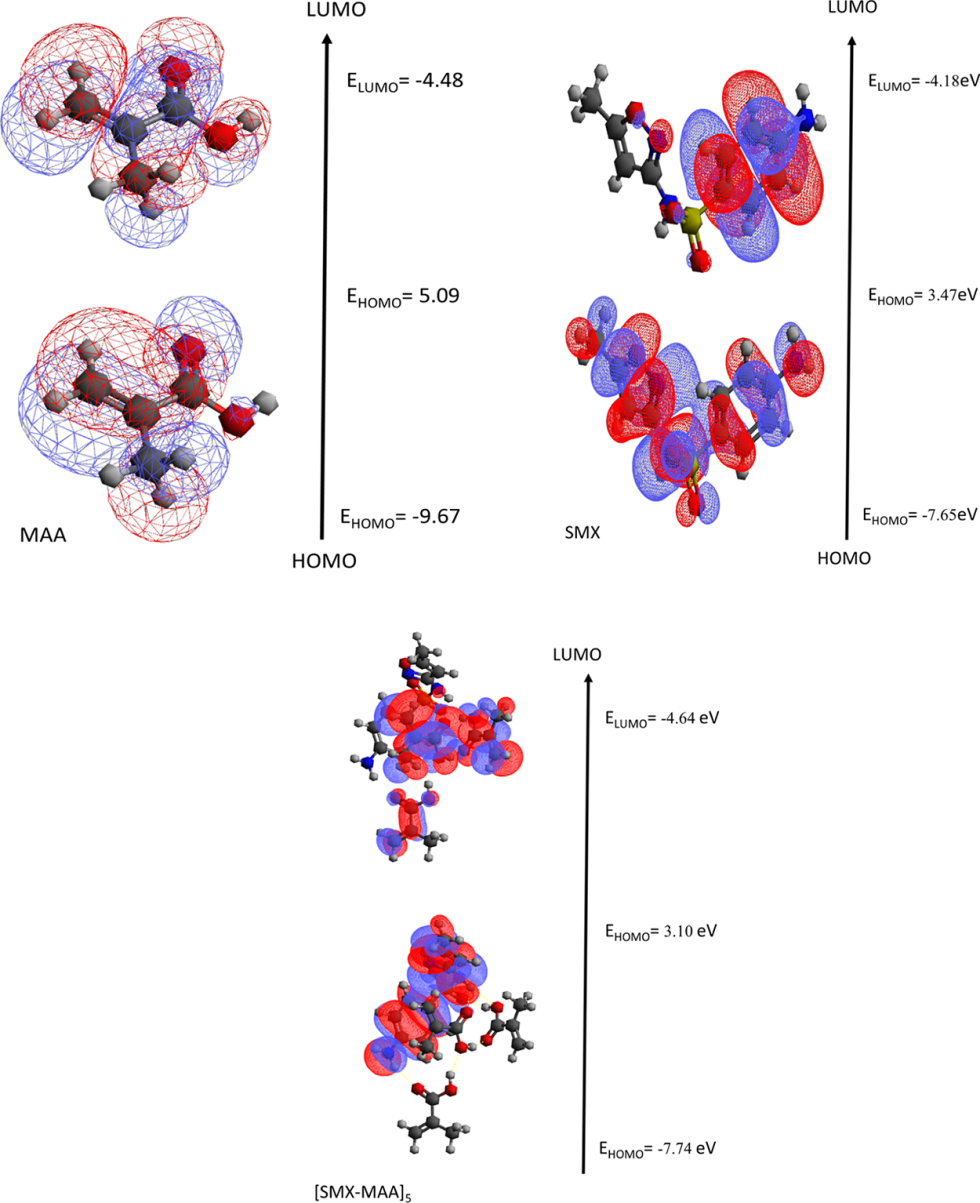
Molecular orbital surfaces and HOMO–LUMO energies (in eV) of the template molecule SMX, functional monomer MAA, and [SMX–MAA]_5_ complexes. Comparison of theoretical FTIR spectrum of MAA and SMX molecules.

It was found that the HOMO energy (−7.65 eV) of the SMX molecule (*E*_HOMO_) in the vacuum was significantly more than the HOMO energy of the MAA molecule (−9.66) (Table 7). It was found that the LUMO energy of the MAA molecule (−4.58 eV) was lower than the LUMO energy of the SMX molecule (−4.18). *E*_Homo_ energy of [SMX–MAA]_5_ complexes was found to be −7.74 and *E*_Lumo_ energy to be −4.64. The HOMO–LUMO energy gap is 3.10 eV. The orbital surfaces of the molecules are given in Figure 6.

**Figure 7:**
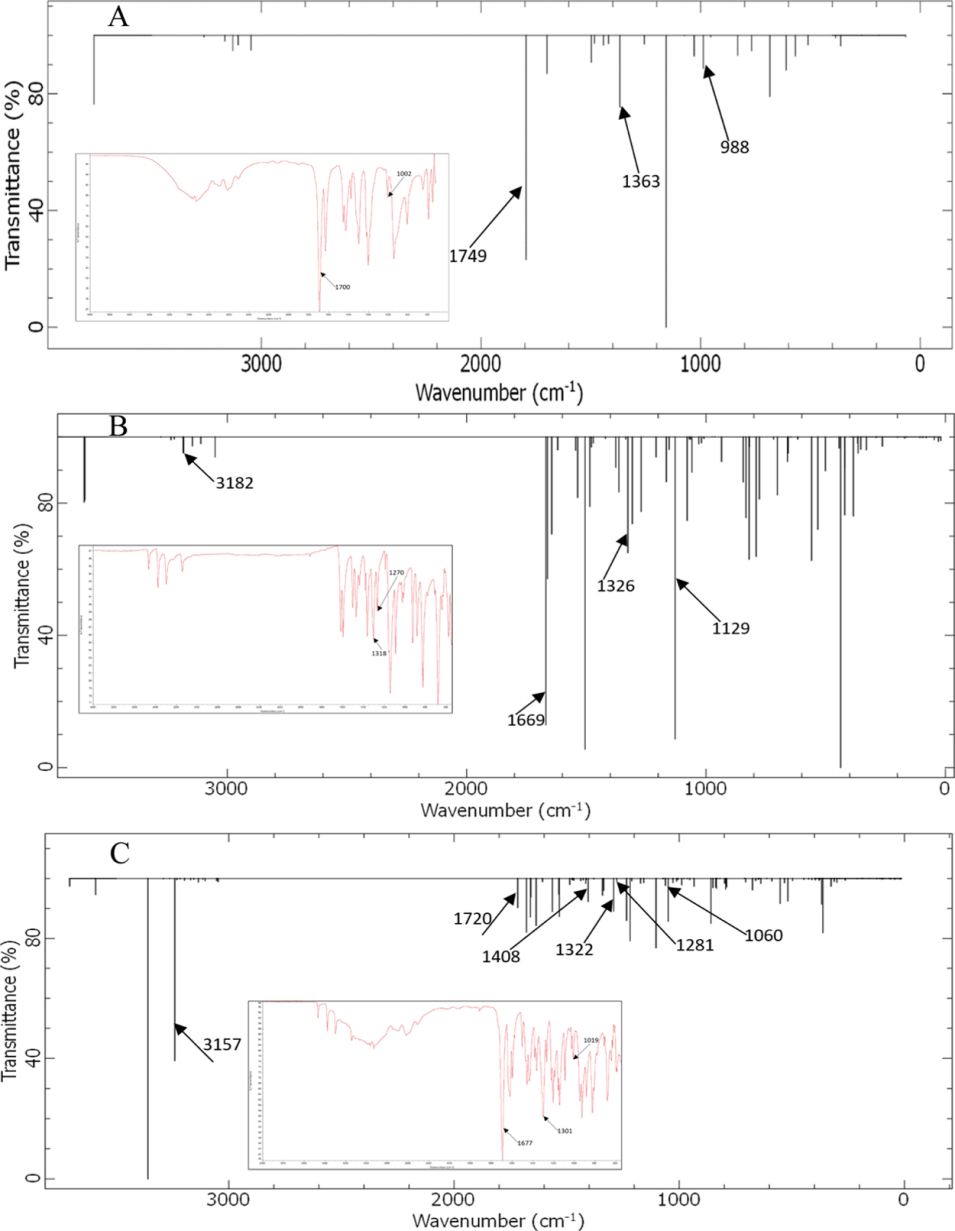
Theoretical and experimental FTIR spectra of the (A) MAA, (B) SMX, and (C) [SMX–MAA]_2_ complexes.

In the theoretical spectrum of the MAA molecule, the peaks at 1749, 1363, and 988 cm^–1^ can be attributed to C=O stretching, O–H in-plane bending, and O–H out-of-plane bending vibrations, respectively. The characteristic vibrations of NH stretching in sulfonamide (3182 cm^–1^), C=N imine stretching (1669 cm^–1^), and S=O stretching (1326 and 1129 cm^–1^) were observed in the theoretical spectrum of SMX. Additionally, based on the theoretical spectrum of the SMX–MAA complex, the N–H stretching and S=O stretching in sulfonamide together with C=O stretching in MAA were shifted to shorter wavenumbers (N–H_str_; 3182–3157 cm^–1^, S=O_str_; 1326–1322 and 1292–1281 cm^–1^, and C=O_str_; 1749–1720 cm^–1^). While O–H in-plane and out-of-plane bending vibrations of MAA were shifted to higher wavenumbers (O–H in-plane bending; 1363–1408 cm^–1^ and O–H out-of-plane bending; 988–1060 cm^–1^). This redshift of stretching vibrations for the related functional groups and the blueshift of bending vibrations strongly support the formation of hydrogen bonds between the corresponding groups. At the same time, the experimental data showed a similar trend with a redshift of C=O (1700–1677 cm^–1^) and S=O (1318–1301 cm^–1^) stretching together with a blueshift of the O–H out-of-plane bending (1002–1019 cm^–1^) and O–H in-plane bending (1270–1301 cm^–1^) (Figure 7).

## Conclusions

Within the scope of this study, the solvent environment and monomer ratio, in which the interactions between SMX and MAA molecules are the most stable, were investigated. The stability of the interactions between the template molecule and the functional monomer is determined by concepts such as hydrogen bonds, interaction energies, Gibbs free energies, and solvation energies. For this purpose, primarily, it was investigated in which region the hydrogen bond interactions between the two molecules were more stable. The complex geometry with the lowest calculated interaction energy and Gibbs energy was the [SMX–MAA]_2_ complex.

After determining the binding site, the interaction energies and intermolecular hydrogen bonds were investigated by increasing the number of monomers in the region where the most stable complex geometry was formed. As a result of the calculations, the ratio of the monomer with the lowest interaction energy and the shortest hydrogen bond length was determined to be one SMX molecule and three MAA molecules. The interaction energy of the complex calculated from the same region in the gas phase at a 1:3 M ratio was calculated to be −37.94 kcal mol^–1^.

This study aimed to establish a theoretical basis for MIP applications, and the choice of the solvent in MIP applications is of great importance. In the synthesis of MIPs, a solvent with a high solvation value weakens the interactions at the interaction sites of the template molecule with the functional monomer. Therefore, the molecular recognition ability is weakened. Molecular geometries and solvation energies were calculated at a 1:1 mole ratio in ethanol, acetonitrile, and DMSO solvents to find the solvent environment where the most stable complex will form between SMX and MAA. Within the scope of the study, the low solvation energy and Gibbs free energy values of the [SMX–MAA]_2_ complex in the acetonitrile environment indicates that the intermolecular interaction is at a maximum level in the acetonitrile environment. The solvation energy of the complex was calculated to be −20.39 kcal mol^–1^ in this solvent at a molar ratio of 1:1.

After determining the correct molar ratio and solvent environment, the [SMX–MAA]_5_ complex, in which the monomer binds to the template molecule at sites with different hydrogen bond potentials, was investigated in a vacuum environment.

The interaction energy of the complex was calculated to be −39.73 kcal mol^–1^. The HOMO–LUMO energy difference was determined to be 3.10 eV. In addition to the theoretical studies, the theoretical FTIR spectra of the SMX, MAA, and SMX–MAA complex in a DMSO environment were compared with the experimental FTIR spectra.

As a result of the study, it was found that the complex geometry that will form between SMX and MAA will be the most stable at a 1:3 M ratio in an acetonitrile solvent. The results of the computational studies of these molecules, which can be used to remove environmental pollution or in drug designs, are a pioneer for the experimental MIP studies.

## Supporting Information Available

Optimized geometry of the most stable compound [SMX–MAA]_2_, optimized geometry of the three monomer complex, optimized geometry of the [SMX–MAA]_5_ complexes in acetonitrile, and IR intensity values (PDF)

## Notes

A further request for data and resources should be directed to and fulfilled by the corresponding author. Software used in the study: Visualization tools; Avogadro v1.2 (http://avogadro.cc/), Gaussview5.0 (https://gaussian.com/). All DFT studies made with Gaussian09 (https://gaussian.com/).
