# Supplementary material for: Computational Investigation of the Monomer Ratio and Solvent Environment for the Complex Formed between Sulfamethoxazole and Functional Monomer Methacrylic Acid
Source: ACS Omega. 2022 May 10;7(20):17175–84. doi: 10.1021/acsomega.2c00862 (PMC9134257; doi:10.1021/acsomega.2c00862)
Supplement: Supplementary file 1 — ao2c00862_si_001.pdf [file ao2c00862_si_001.pdf]

Computational Investigation of the Monomer Ratio and Solvent Environment for the Complex Formed Between Sulfamethoxazole and Functional Monomer Methacrylic Acid

Sisem Ektirici[1], Önder Kurç[1], Mitra Jalilzadeh[1], Süleyman Aşır[2], Deniz Türkmen[1*]

[1]*Department of Chemistry, Faculty of Science, Hacettepe University, Beytepe, Ankara, Turkey*

[2]*Department of Materials Science and Nanotechnology Engineering, Near East University, Nicosia, Mersin 10 Turkey, North Cyprus*

*Corresponding Author: denizt@hacettepe.edu.tr

Table S1. Optimized geometry structure coordinates of [SMX-MAA]2 complex in gas

Atom	X	Y	Z
H	0.5341	-3.46831	-2.02387
H	2.64299	-4.06436	-3.02271
H	-0.29115	3.31477	-0.80486
C	1.10212	-2.90031	-1.29876
H	-0.62979	-2.05662	-0.36598
H	1.52715	5.1019	-2.04494
C	0.45088	-2.11104	-0.37512
H	3.04502	4.24584	-2.36904
C	0.67117	2.96432	-0.4796
N	3.1489	-3.81196	-2.19086
C	2.37181	4.59495	-1.58672
H	-0.91944	1.01574	0.49243
C	2.50069	-2.98395	-1.29878
C	1.89733	3.4531	-0.76816
N	0.00054	1.03916	0.94613
C	0.95908	1.8589	0.37217
H	4.12414	-3.62503	-2.35111
C	1.19108	-1.40055	0.56157
O	-0.96087	-0.94894	1.96563
O	2.83603	2.71957	-0.15986
N	2.23795	1.70361	0.56418
H	2.91771	5.30423	-0.9652
S	0.35864	-0.37662	1.72208
C	3.22629	-2.25908	-0.34712

C	2.5774	-1.46769	0.57826
H	4.30647	-2.32338	-0.3396
H	3.12837	-0.895	1.31022
O	1.23123	-0.11066	2.82835
H	-6.02362	-0.80497	0.62936
C	-4.73673	0.09265	-0.82842
C	-4.89787	1.01812	-1.76537
C	-5.7793	-0.90557	-0.42804
C	-3.41939	0.04981	-0.12327
O	-3.3551	-0.90741	0.78177
O	-2.5014	0.81206	-0.35986
H	-5.8275	1.10478	-2.31182
H	-2.47598	-0.8935	1.23528
H	-6.68397	-0.76727	-1.01595
H	-4.09639	1.70565	-1.997
H	-5.41453	-1.92292	-0.56868

Table S2. Optimized geometry structure coordinates of [SMX-MAA]2 complexes in acetonitrile

Atom	x	y	z
H	3.01423	-3.87002	-3.04907
H	-6.06762	1.06282	-2.02644
N	3.44674	-3.63728	-2.1692
H	4.44909	-3.53579	-2.19241
H	2.84897	4.42306	-2.32126
C	-5.11538	0.98374	-1.51764
H	0.84915	-3.1955	-2.26249
H	-4.40781	1.7944	-1.63054
H	1.27483	5.18206	-2.01484
C	2.14328	4.72577	-1.54739
C	1.3523	-2.71372	-1.43428
C	2.74523	-2.86258	-1.29546
H	-6.6697	-1.09119	-1.10984
C	-4.8236	-0.07683	-0.77334
C	1.72508	3.55259	-0.74866
C	-5.74154	-1.24109	-0.56194
H	2.63352	5.46231	-0.91012
H	-0.44973	3.29438	-0.82581
H	-5.27821	-2.16863	-0.90118
C	0.52383	2.98855	-0.48675

C	0.6362	-1.96827	-0.52621
O	-2.63579	0.75275	-0.2528
C	3.39172	-2.23406	-0.21756
C	-3.49313	-0.09803	-0.09958
H	-0.43561	-1.86289	-0.64131
H	4.46299	-2.34328	-0.10802
O	2.69487	2.86406	-0.13023
C	0.85766	1.88955	0.35297
C	1.29743	-1.35995	0.53914
C	2.67413	-1.48517	0.69008
N	2.14146	1.80384	0.57098
H	-5.97435	-1.36867	0.4958
H	-0.98862	0.95973	0.48238
O	-3.32827	-1.14164	0.69532
N	-0.05377	1.00464	0.90592
H	-2.4404	-1.09465	1.11911
H	3.17314	-0.99391	1.51359
S	0.37832	-0.39528	1.6706
O	-0.911	-1.03826	1.9101
O	1.2018	-0.07831	2.80991

Table S3. Optimized geometry structure coordinates of the [SMA-MAA]5 complex in gas

Atom	X	Y	Z
O	-2.4133	-1.92527	-2.67887
O	-0.0135	-1.31399	-2.28933
S	-1.41871	-1.23811	-1.90772
H	3.67352	0.39432	-0.36364
C	-1.54012	-1.71077	-0.21858
H	-3.58118	-2.32536	-0.39558
N	-1.71459	0.39514	-1.99878
O	4.34962	1.12128	-0.41119
C	-2.73597	-2.22179	0.26986
C	-0.43189	-1.56126	0.60733
H	0.4894	-1.14202	0.22228
N	-3.99844	0.22636	-1.36355
H	-0.93301	0.9237	-1.61515
C	-2.94842	0.94177	-1.64881
C	-2.82138	-2.57892	1.60049
C	3.86737	2.24947	0.0718
O	2.76359	2.35412	0.56634

C	-0.51796	-1.93591	1.93093
H	-3.74821	-2.97592	1.99415
C	-1.71866	-2.43431	2.45018
H	6.69929	2.34956	-0.13798
O	-4.9946	1.14372	-1.08542
H	0.34398	-1.82573	2.57683
C	-3.20797	2.339	-1.56311
C	4.82776	3.38956	-0.05163
N	-1.79911	-2.81509	3.77353
C	6.15784	3.12069	-0.68605
C	-4.50906	2.38726	-1.2067
H	-2.5182	3.14737	-1.71842
O	-0.67295	1.75295	0.2818
H	6.03186	2.74972	-1.70316
H	1.41523	1.59903	1.32465
H	-2.72152	-2.8787	4.16963
H	-1.11556	-2.42019	4.39728
C	4.43597	4.57055	0.40862
H	3.45639	4.68568	0.8515
C	-0.48417	1.44489	1.43857
H	-6.29761	3.45402	-1.61955
O	0.7213	1.3362	1.97457
H	-3.02924	1.19232	0.91376
H	6.75876	4.02711	-0.71006
C	-5.45191	3.4985	-0.93398
C	-2.81391	1.03994	1.96151
C	-1.57228	1.13453	2.4193
H	5.08163	5.4367	0.3497
H	-4.9472	4.45298	-1.05458
H	-0.43926	0.13644	3.93978
H	-5.83793	3.42656	0.08254
H	-3.63556	0.79516	2.62214
C	-1.18726	0.925	3.85239
H	-2.06087	0.65989	4.44541
H	-0.736	1.82576	4.26994
H	3.52367	-4.81027	-0.74092
H	4.33995	-4.08956	-2.10928
H	5.2805	-4.5698	-0.68406
C	4.33846	-4.1429	-1.0207
O	2.06092	-2.75809	-1.50453

C	4.13762	-2.78438	-0.42207
H	1.2689	-2.21889	-1.76762
C	2.89335	-2.04342	-0.78755
H	5.89773	-2.69924	0.71804
C	4.98349	-2.20353	0.41996
O	2.65347	-0.89288	-0.45263
H	4.78327	-1.2239	0.831

Table S4. FTIR of MAA

Frequencies	Intensities
66.6263	0.431154
188.91	0.102116
267.697	0.107588
362.881	3.53969
385.159	0.811695
511.071	3.22671
568.885	7.06064
610.293	11.8555
684.91	20.9708
768.168	5.21182
831.672	6.81002
953.676	0.499132
987.816	11.3269
1029.58	7.09731
1074.23	0.0376468
1157.12	100
1256.46	2.93841
1367.75	24.6145
1419.05	2.78649
1442.17	3.3037
1483.15	2.66629
1497.43	9.211
1698.89	13.0429
1794.41	76.805
3046.86	5.01779
3105.35	3.2436
3130.1	5.16198
3166.42	1.9042
3261.55	0.519145
3762.65	23.5465

Table S5. FTIR of SMX

Frequencies	Intensities
19.454	1.0961
29.3617	1.3465
46.1513	0.777179
80.4186	0.248059
95.4221	0.215162
106.4	0.179439
161.871	0.101105
170.298	0.351816
199.444	0.132039
263.087	2.79131
285.871	0.0525331
289.417	0.175237
329.85	3.90102
353.458	3.75124
364.71	4.86232
365.727	1.9091
384.539	23.8757
419.218	9.14926
420.409	23.5582
437.704	100
443.915	3.36594
501.215	10.2037
532.916	27.9669
558.994	37.3672
616.856	0.667359
646.078	0.101759
656.375	4.85812
659.688	7.42239
701.764	17.5068
717.915	0.702703
776.898	18.7337
790.547	36.1263
818.831	2.89176
819.82	36.9891
833.073	24.4053
844.303	13.5914
935.148	7.40978
963.201	0.0629363
974.953	0.031382
1008.8	0.439417

1019.19	1.79784
1030.04	2.08176
1058.83	10.6297
1064.38	0.71035
1070.29	0.638905
1078.88	25.2628
1129.16	91.3336
1154.06	3.76808
1165.84	13.5263
1208.69	6.05673
1271.2	22.546
1307.92	26.2516
1326.29	35.0308
1335.35	0.686891
1363.96	16.5786
1377.02	9.14923
1420.23	0.416131
1470.37	1.85957
1478.64	3.05891
1485.42	21.0181
1505.25	94.4644
1536.4	18.2602
1544.46	4.03299
1619.58	3.89864
1644.47	29.3507
1662.4	42.8921
1668.92	87.0455
3050.77	6.04823
3110.66	2.02523
3146.27	2.69083
3182.52	4.85998
3185.31	4.73704
3221.81	0.661744
3235.69	0.839254
3277.64	0.0896335
3595.78	18.9749
3598.28	19.7257
3709.48	8.87702

Table S6. FTIR of [SMX-MAA]2

Frequencies	Intensities
13.0203	0.100069

15.294	0.141926
20.6203	0.209531
30.5351	0.0751671
41.3297	0.171739
44.3367	0.218864
62.1474	0.205454
67.8552	0.0203417
88.6868	0.00409779
97.7491	0.2791
103.805	0.0166693
110.085	0.0671842
122.888	0.136221
156.738	0.041894
186.304	0.0207098
189.08	0.0244191
209.631	0.127755
263.217	0.60867
288.521	0.0610089
300.041	1.28611
312.244	0.992079
325.989	2.61451
362.075	18.1006
368.195	8.591
375.635	0.369619
379.552	0.630782
395.694	0.490001
396.508	0.370323
411.194	0.410642
425.457	0.214218
447.014	0.28193
496.86	0.713229
517.774	7.53104
532.714	1.40434
552.124	8.33575
588.798	0.85138
611.616	0.0729055
637.545	1.64734
643.802	0.0823157
649.609	0.356627
660.873	0.692179
674.776	3.81013
704.89	1.15911

710.137	0.0133362
789.454	2.77896
793.668	3.5849
798.626	1.5914
819.815	0.0340583
830.785	0.0232617
834.843	3.30909
838.141	2.87918
850.649	3.03724
859.408	14.9541
934.409	2.60445
953.301	0.442684
965.066	0.0152297
979.278	0.0139415
989.302	2.15466
1006.39	0.349261
1011.86	0.891798
1027.85	1.35259
1028.72	0.408839
1048.91	14.3135
1061.13	2.32753
1062.12	0.164206
1071.57	0.0242882
1071.73	0.147823
1103.28	23.1187
1157.24	1.3702
1171.76	1.54608
1210.89	0.828065
1218.19	20.7897
1233.89	13.9993
1277.25	0.904696
1291.29	10.928
1335.29	3.97934
1340.81	5.58825
1378.76	0.166484
1404.49	7.70304
1412.82	0.472489
1415.27	1.61823
1426.55	0.554057
1437.19	0.769432
1467.83	0.60923
1472.27	0.549354

1474.32	0.235186
1476.52	0.333349
1486.18	2.0125
1532.42	12.6904
1534.29	5.29801
1563.52	11.0246
1607.09	1.08098
1635.39	15.6848
1659.3	6.18635
1660.82	12.8168
1678.4	17.8959
1717.08	9.72534
3047.47	1.07767
3054.25	0.736593
3105.25	0.748064
3115.12	0.335066
3135.2	1.1999
3155.83	0.344243
3170.05	0.444778
3197.38	0.61244
3197.98	0.573335
3217.42	0.160415
3231.42	0.406266
3240.42	60.8078
3263.65	0.0520362
3291.46	0.126406
3359.75	100
3592.12	5.3659
3707.18	2.55413
